# Supplementary material for: The B. subtilis replicative polymerases bind the sliding clamp with different strengths to tune their activity in DNA replication
Source: Nucleic Acids Res. 2025 Jul 30;53(14):gkaf721. doi: 10.1093/nar/gkaf721 (PMC12309361; doi:10.1093/nar/gkaf721)
Supplement: gkaf721_Supplemental_File [file gkaf721_supplemental_file.pdf]

“The *B. subtilis* replicative polymerases bind the sliding clamp with different strengths to tune their activity in DNA replication”

Luke G. O’Neal,[1] Madeline N. Drucker,[1] Ngoc Khanh Lai,[2] Ashley F. Clemente,[1] Alyssa P. Campbell,[1] Lindsey E. Way,[2] Sinwoo Hong,[1] Emily E. Holmes,[1] Sarah J. Rancic,[1] Nicholas Sawyer[1]*, Xindan Wang[2]*, Elizabeth S. Thrall[1]*

[1]Department of Chemistry and Biochemistry, Fordham University, Bronx, NY 10458

[2]Department of Biology, Indiana University, Bloomington, IN 47405

*****Correspondence: ethrall@fordham.edu, xindan@iu.edu, nsawyer@fordham.edu

SUPPLEMENTARY INFORMATION

This PDF file includes:

Figures S1 to S9
Tables S1 to S14
Supplementary Methods
Supplementary References

Supplementary Figures

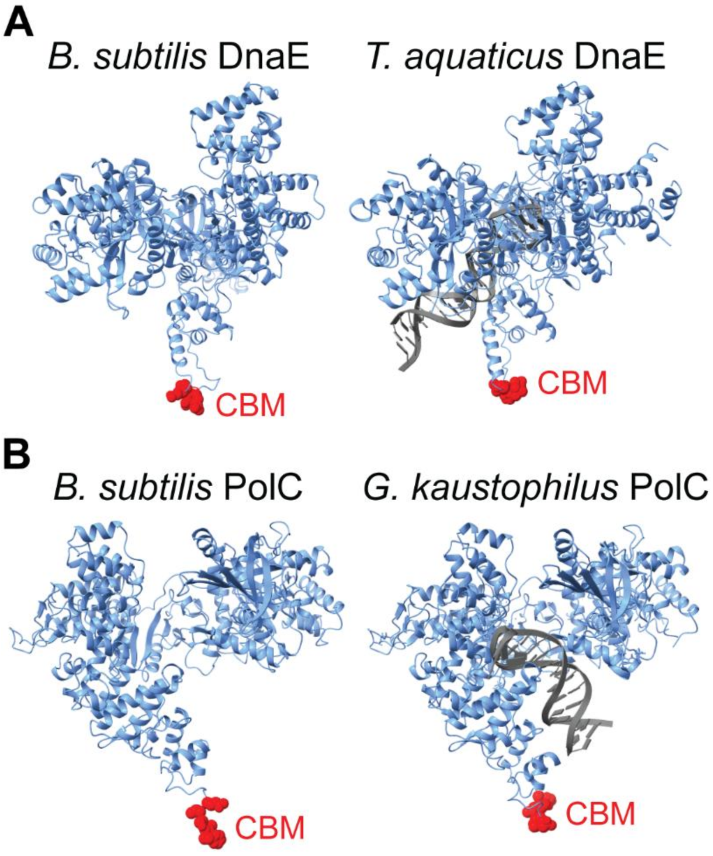

Figure S1. *B. subtilis* polymerase structures and comparisons to homologs. (A) AlphaFold predicted structure of *B. subtilis* DnaE (AlphaFold DB: AF-O34623-F1) (left) and experimental crystal structure of *T. aquaticus* DnaE (PDB: 3E0D)(1) (right). (B) AlphaFold predicted structure of *B. subtilis* PolC (AlphaFold DB: AF-P13267-F1) (left) and experimental crystal structure of *G. kaustophilus* PolC (PDB: 3F2B)(2) (right). The experimental PolC structure contains truncations of the N-terminal domain (residues 1 – 232) and exonuclease domain (residues 413 – 616). The corresponding truncations were made to the AlphaFold structure for comparison. CBMs are highlighted in red.

CBM	Analytical HPLC Chromatogram
**QLSLPL**	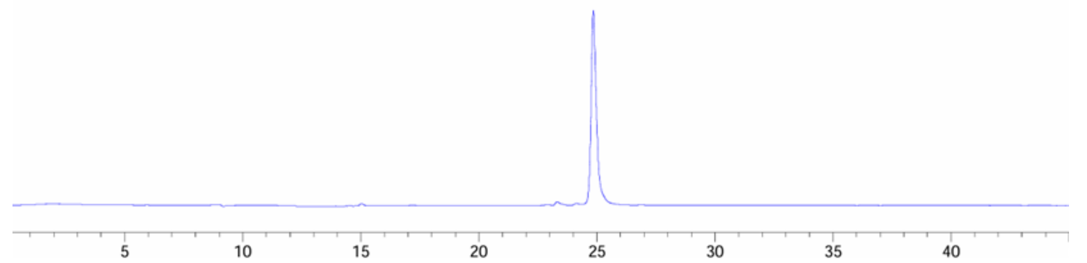
**QADMA**	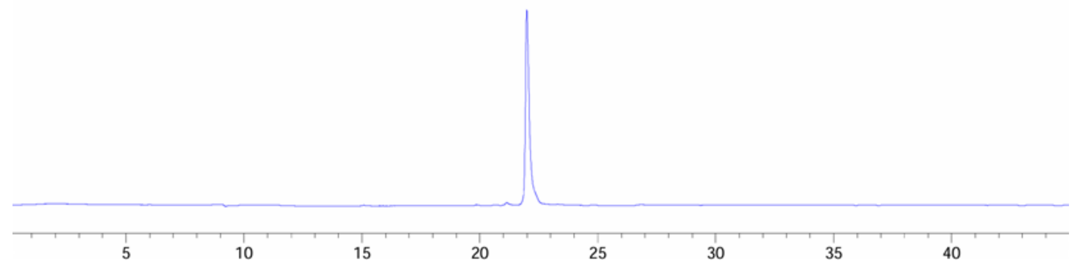
**QLSLF (PolC context)**	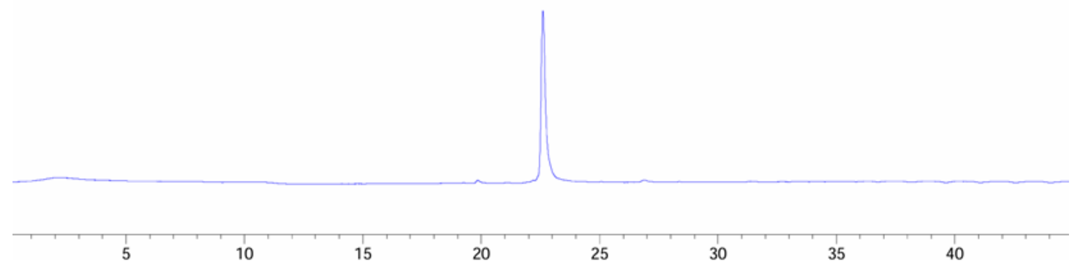
**QMGLF**	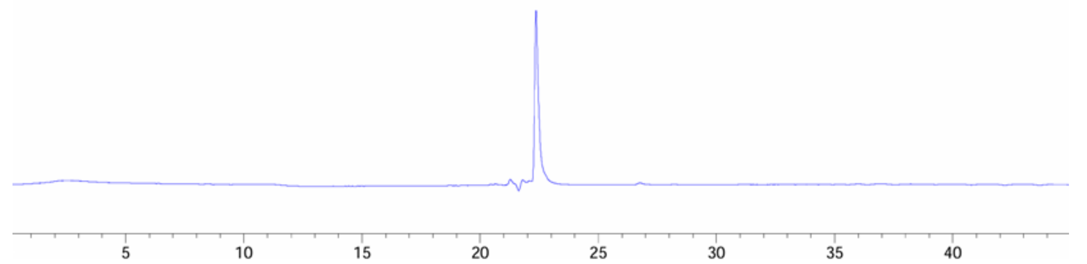
**AMGLA**	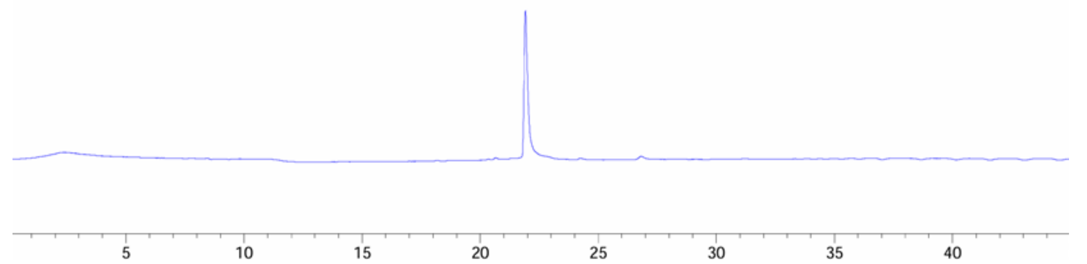
**QLSLF (DnaE context)**	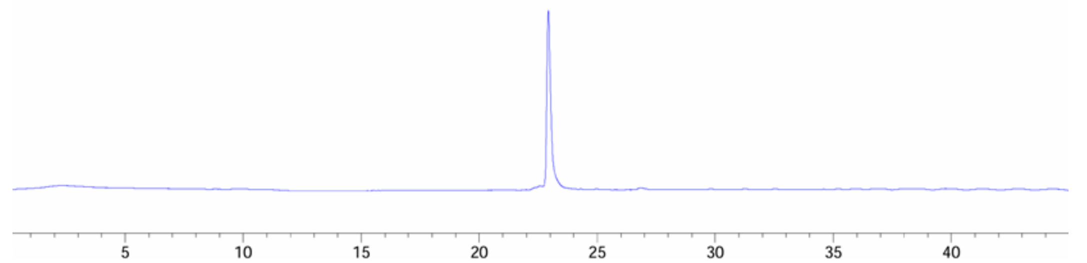

Figure S2. Analytical HPLC chromatograms for all peptides, measured at 280 nm.

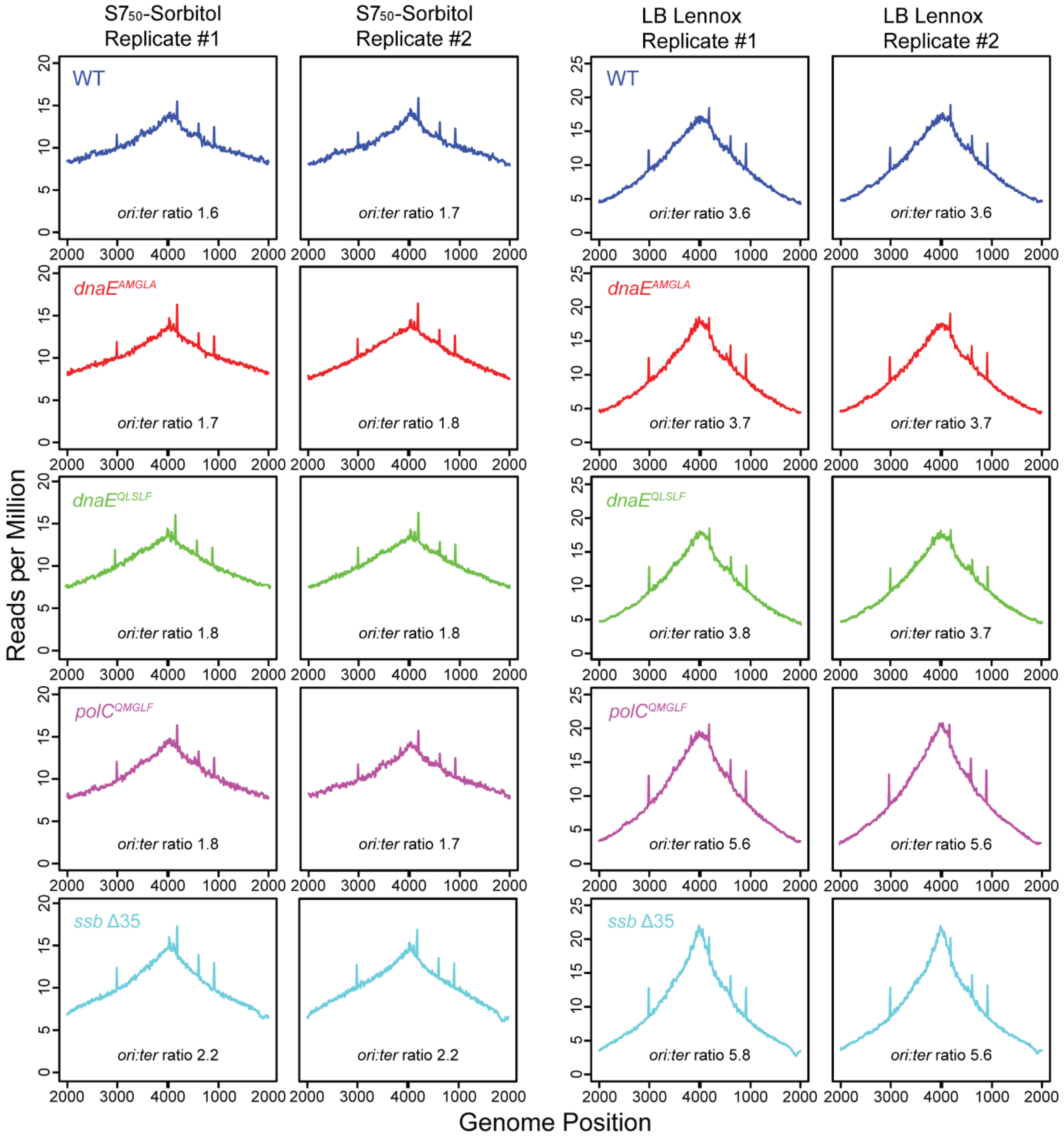

Figure S3. Whole-genome sequencing analysis for WT and CBM mutant strains of DnaE and PolC. Individual plots showing the normalized number of reads vs. genome location for (top to bottom) WT, *dnaE*^{AMGLA}, *dnaE*^{QLSLF}, *polC*^{QMGLF}, and *ssb* Δ35 strains in (left) S7$_{50}$-sorbitol and (right) LB Lennox media, respectively.

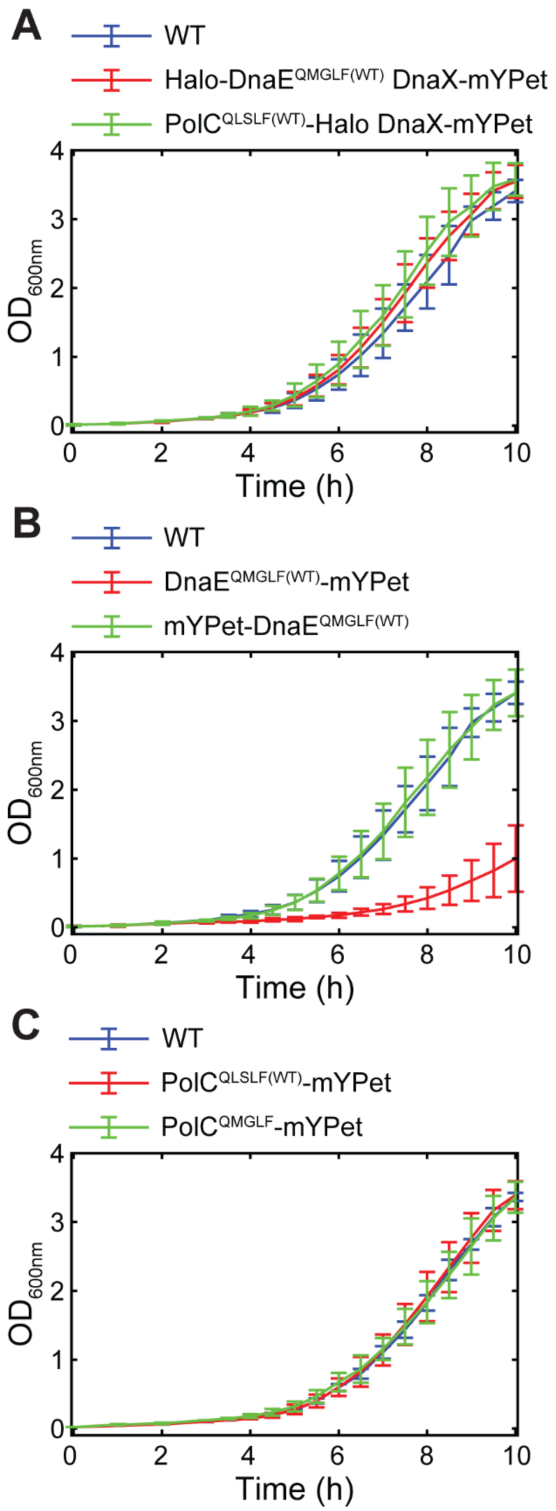

Figure S4. Liquid culture growth curves for imaging strains in S7$_{50}$-sorbitol media. (A) WT, Halo-DnaE$^{QMGLF(WT)}$ DnaX-mYPet, and PolC$^{QLSLF(WT)}$-Halo DnaX-mYPet. (B) WT, DnaE$^{QMGLF(WT)}$-mYPet, and mYPet-DnaE$^{QMGLF(WT)}$. (C) WT, PolC$^{QLSLF(WT)}$-mYPet, and PolC^{QMGLF}-mYPet.

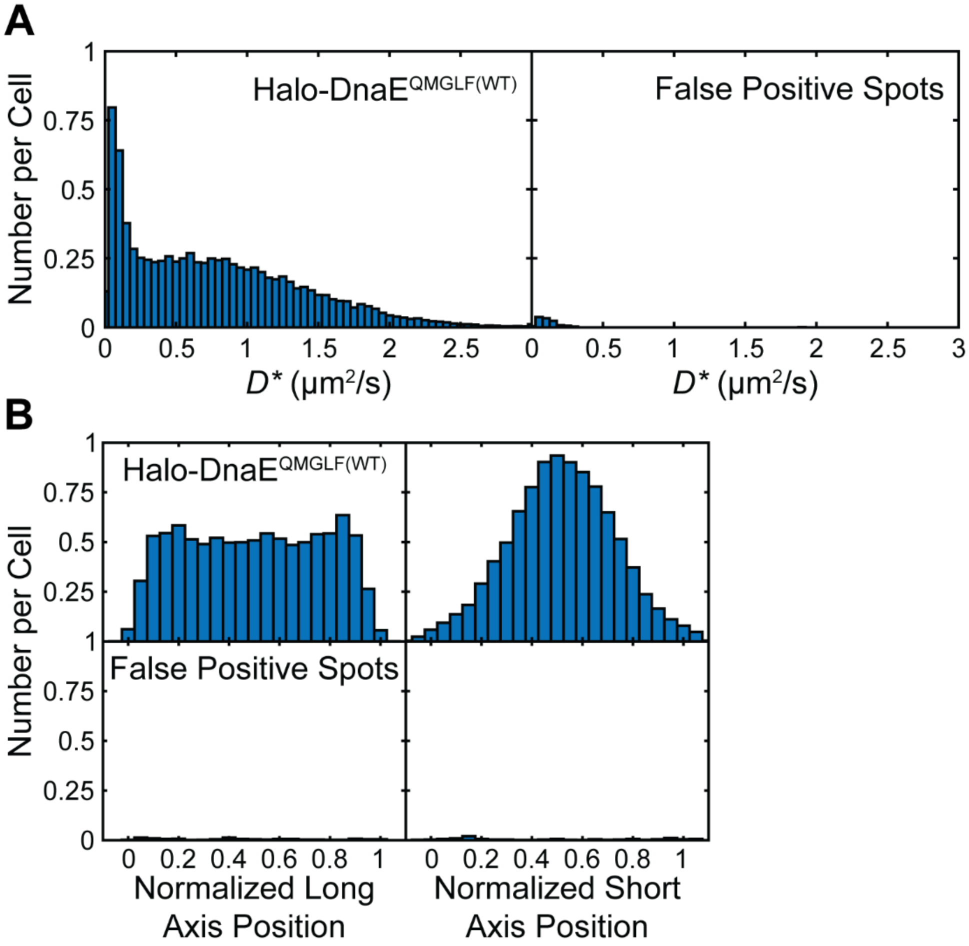

Figure S5. Comparison of false positive signal to Halo-DnaE signal. (A) Apparent diffusion coefficient (D^*) distributions for (left) Halo-DnaE$^{QMGLF(WT)}$ and (right) false positive spots on a per cell basis. (B) Average cellular localization projected along normalized long and short cell axes for (top) Halo-DnaE$^{QMGLF(WT)}$ and (bottom) false positive spots on a per cell basis. The plotted data represent at least two (false positive spots) or three (Halo-DnaE$^{QMGLF(WT)}$) independent replicates (see Table S5).

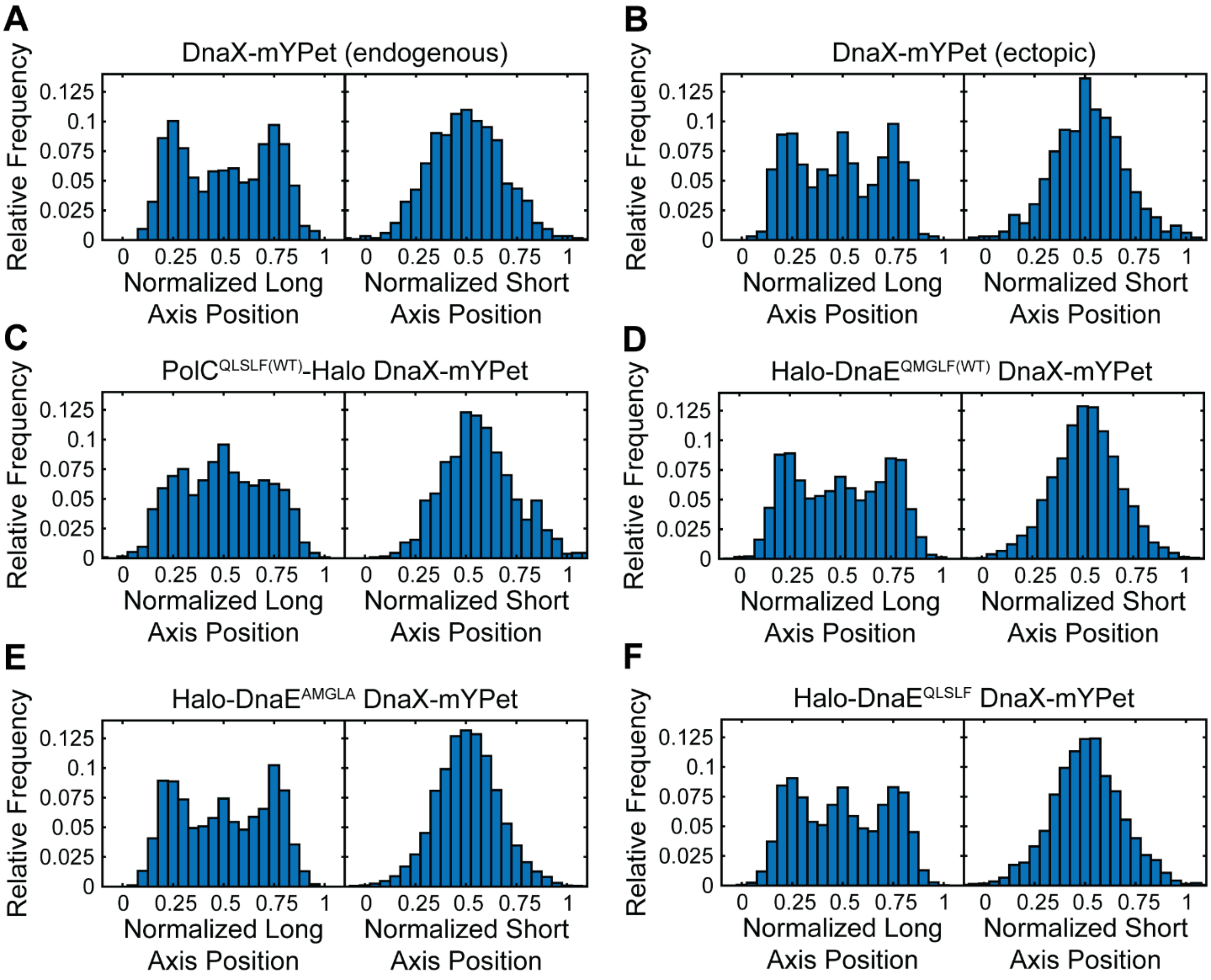

Figure S6. Average cellular localization of DnaX-mYPet foci projected along normalized long and short cell axes. (A) Endogenous DnaX-mYPet. (B) Ectopic DnaX-mYPet. (C) PolC-Halo DnaX-mYPet. (D) Halo-DnaE$^{QMGLF(WT)}$ DnaX-mYPet. (E) Halo-DnaE^{AMGLA} DnaX-mYPet. (F) Halo-DnaE^{QLSLF} DnaX-mYPet. The plotted data represent at least three independent replicates (see Table S5).

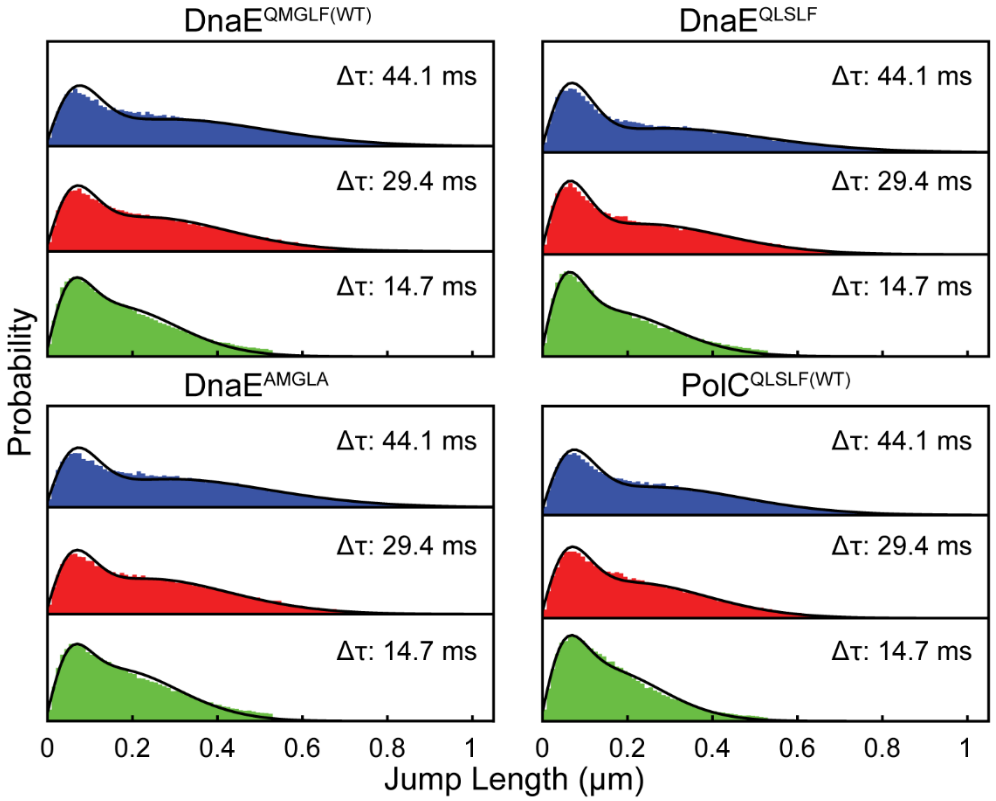

Figure S7. Spot-On diffusion analysis for Halo-DnaE and PolC-Halo, showing jump length distributions and corresponding two-population fits. (Top left) Halo-DnaE$^{QMGLF(WT)}$, (bottom left) Halo-DnaE^{AMGLA}, (top right) Halo-DnaE^{QLSLF}, and (bottom right) PolC$^{QLSLF(WT)}$-Halo. The plotted data represent at least three independent replicates (see Table S5).

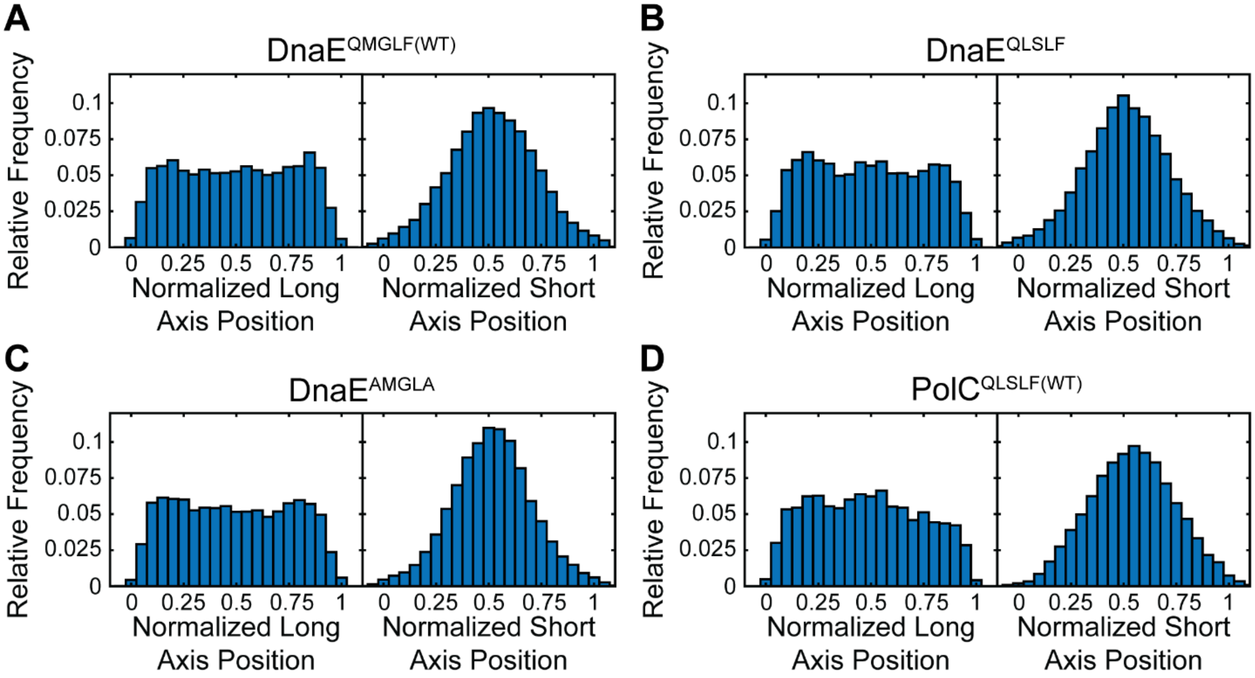

Figure S8. Average cellular localization of single Halo-DnaE or PolC-Halo molecules projected along normalized long and short cell axes. (A) Halo-DnaE$^{QMGLF(WT)}$. (B) Halo-DnaE^{QLSLF}. (C) Halo-DnaE^{AMGLA}. (D) PolC$^{QLSLF(WT)}$-Halo. The plotted data represent at least three independent replicates (see Table S5).

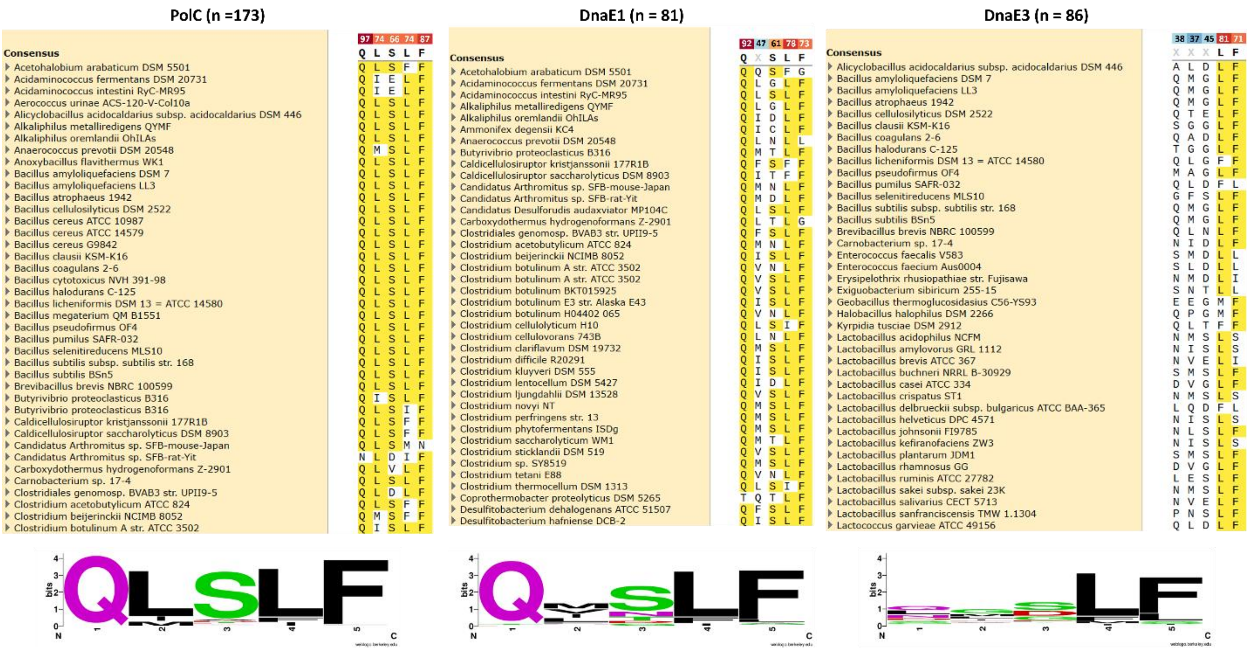

Figure S9. Multiple sequence alignment of PolC, DnaE1, and DnaE3 polymerase CBM sequences from Firmicutes species. CBM data were extracted from the representative polymerase dataset of Timiniskas, et al.(3) For each group, the consensus CBM sequence and percent conservation at each CBM position are displayed along with an alignment excerpt (residues highlighted in yellow if they match the consensus and appear in > 50% of the aligned sequences) and sequence logo.

Supplementary Tables

Table S1. Oligonucleotides used in this study. (Lowercase letters indicate bases that do not prime on the template but provide homology for Gibson assembly of PCR fragments.)

Number	Designation	Sequence (5′-3′)
oEST082	Ec-pET28b-his6-dnaN-for	ggcagcagccaccatcaccaccatcatagcagcggcATGAAATTTACCGTAGAACG
oEST083	Ec-pET28b-dnaN-rev	gctttgttagcagccggatcTTACAGTCTCATTGGCATGA
oEST084	Bs-pET28b-his6-dnaN-for	ggcagcagccaccatcaccaccatcatagcagcggcATGAAATTCACGATTCAAAA
oEST085	Bs-pET28b-dnaN-rev	gctttgttagcagccggatcTTAATAGGTTCTGACAGGAA
oEST086	pET28b-his6-for	GATCCGGCTGCTAACAAAGC
oEST087	pET28b-his6-rev	tggtgatggtggctgctgccCATGGTATATCTCCTTCTTAAAG
oEST118	yvbJ-upstream-for	CTTCCTGTACCGATGATGAACATC
oEST119	yvbJ-Nter-rev	TTTAAGTCATAATGGTCAAATACCAGC
oEST120	yvbJ-Nter-cat-iso-for	tttgaccattatgacttaaaCCATACGGCAATAGTTACCCTTAT
oEST121	dnaX-yvbJ-Cter-iso-rev	gaaaatccactttttctgttGAATCGACAGAAGGTGCACGGTAA
oEST122	yvbJ-Cter-for	AACAGAAAAAGTGGATTTTCGAAG
oEST123	yvbJ-downstream-rev	AATAGGAAGGACGTATACAGATGT
oML83	sequencing oligo in *kan*	CCTCATCCTCTTCATCCTC
oWX225	region at C-ter of *polC*	GACTTGATGCTTCACCGAAGG
oWX228	region at C-ter of *polC*	CGCTCTAGAGCTTACTTTGGCACATTCCTC
oWX323	region downstream of *polC*	GGTCCCTGTCCTGATCCCTCGAGGAACAGTGACAATTGGTTTTG
oWX324	region downstream of *polC*	CGCGGGATCGAGATCTGCATTAATAATATGGAAAGCAGAATTTCTC
oWX438	universal oligo for *loxP* cassettes	GACCAGGGAGCACTGGTCAAC
oWX439	universal oligo for *loxP* cassettes	TCCTTCTGCTCCCTCGCTCAG
oWX900	region upstream of *dnaE*	TCTCGTATTGCCTTCGAGTATGCCGCG
oWX902	sequencing *dnaE*	GCGGACCTATTGAGAAGGGCGGTCAGC
oWX903	region upstream of *dnaE*	CTGAGCGAGGGAGCAGAAGGATCCGTATAACAAAAAGCGAATCGTTACC
oWX904	region downstream of *dnaE*	GGTAGTTGACCAGTGCTCCCTGGTCAATTGGAGCAATTATGTGATCCTG
oWX905	region downstream of *dnaE*	TCACCTCCTGTTTGATGCATATCCAGC TCACCTCCTGTTTGATGCATATCCAGC
oWX907	sequencing *dnaE*	AGTTACAACTGCTACCATGTTCCC
oWX916	region upstream of *polC* gene	CAAAAGCTTGCGGAAGACCAAGAGCGG
oWX919	region inside of *polC* gene	GAGCAGAAGGATCCCTGAGAAATTCTGCTTTCCATATTAG

oWX920	region downstream *polC spec* and region downstream *polC mYpet cat*	TGACCAGTGCTCCCTGGTCAAATTCTGCTTCTATGCATACATAAGCG
oWX921	region downstream *polC spec* and region downstream *polC mYpet cat*	CTGATCAGCTTATTCGCAAAACCTTGG
oWX2235	region upstream of *ssb*	CTGATAATGGAATGGGAAGTACGC
oWX2236	region upstream of *ssb*	CTGAGCGAGGGAGCAGAAGGATTAGAATGGAAGATCATCATCCGAG
oWX2238	for *ssb* Δ35	CTGAGCGAGGGAGCAGAAGGATTACCCAAATGGATTATCATTTTGGCC
oWX2239	region downstream of *ssb*	GTTGACCAGTGCTCCCTGGTCTGTGATTATCGCCTAAAATGAAAAAG
oWX2240	region downstream of *ssb*	ATGATACAAATCATTCGCTTCGCC
oWX2241	for *dnaE* Q885A	GATTCATCTAAAAACAATCCCATCGCGTCATCGTCCGCGGCGAATAGC
oWX2242	for *dnaE* Q885A	GCTATTCGCCGCGGACGATGACGCGATGGGATTGTTTTTAGATGAATC
oWX2249	for *dnaE* Q885A	GTCATCGTCCGCGGCGAATAGC
oWX2354	for *dnaE*	CAATCCCATCGCGTCATCGTCCGC
oWX2355	for *dnaE* Q885A F889A	GCGGACGATGACGCGATGGGATTGGCTTTAGATGAATCGTTTTCAATTAAGC
oWX2357	for *dnaE* M886L G887S	GCTATTCGCCGCGGACGATGACCAACTGTCATTGTTTTTAGATGAATCGTTTTC
oWX3633	region upstream of *polC* gene	GAACAATCCCATTTGGTTTTGATCAGGCAGTGAC
oWX3634	for *polC* CBM mutant	AAAACCAAATGGGATTGTTCTAATATGGAAAGCAGAATTTCTC
oWX3692	region downstream *polC mYpet*	AAAACCAAATGGGATTGTTCCTCGAGGGATCAGGACAGGGACC

Table S2. Plasmids used in this study.

Number	Designation	Containing Strain	Source or Reference
pEST005	*pET-28b-mCherry-his*$_6$	EST149	Gift of Joseph Loparo (Harvard Medical School); plasmid pET012
pEST007	*pET-28b-his*$_6$*-Ec-dnaN*	EST199	This study
pEST009	*pET-28b-his*$_6$*-Bs-dnaN*	EST201	This study
pWX318	*mYpet b.s. cat*	cWX514	(4)
pWX340	*dnaX-mYpet b.s.Ω cat*	cWX571	(4)
pWX466	*loxP-spec-loxP*	cWX856	(5)
pWX470	*loxP-kan-loxP*	cWX860	(5)

Table S3. *B. subtilis* bacterial strains used in this study.

Number	Designation or description	Relevant genotype	Construction or source strain designation	Reference
BWX2231	PolC loxP-spec-loxP	*PY79 polC loxP-spec-loxP*	Transformation: *polC loxP-spec-loxP* → EST003	This study
EST001	*E. coli* cloning strain	DH5a	—	—
EST003 (WT)	*B. subtilis* prototrophic wild-type strain	PY79	—	(6, 7)
EST007 (BWX5094)	SSB Δ35	PY79 *ssb Δ35 loxP-kan-loxP*	Transformation: *ssb* D*35 loxP-kan-loxP* → EST003	This study
EST009 (BWX5096)	DnaE loxP-kan-loxP	PY79 *dnaE loxP-kan-loxP*	Transformation: *dnaE loxP-kan-loxP* → EST003	This study
EST013 (BWX5106)	DnaE^{AMGLF}	PY79 *dnaE*^{AMGLF} *loxP-kan-loxP*	Transformation: *dnaE*^{AMGLF} *loxP-kan-loxP* → EST003	This study
EST043 (BWX5217)	DnaE^{AMGLA}	PY79 *dnaE*^{AMGLA} *loxP-kan-loxP*	Transformation: *dnaE*^{AMGLA} *loxP-kan-loxP* → EST003	This study
EST047 (BWX5220)	DnaE^{QLSLF}	PY79 *dnaE*^{QLSLF} *loxP-kan-loxP*	Transformation: *dnaE*^{QLSLF} *loxP-kan-loxP* → EST003	This study
EST051	DnaE-mYPet	PY79 *dnaE-mYpet cat*	Gift of Joseph Loparo (Harvard Medical School); strain ET017	—
EST053 (BWX519)	DnaX-mYPet	PY79 *dnaX-mYpet cat Ω pWX340a*	Strain BWX519 from plasmid pWX340	(4)
EST057 (BWX499)	PolC-mYPet	PY79 *polC-mYPet cat*	Transformation: *polC-mYPet cat* → EST003	This study
EST083	PolC-Halo	PY79 *polC-halo loxP-spec-loxP*	Gift of Joseph Loparo (Harvard Medical School); strain ET096	—
EST085	mYPet-DnaE	PY79 *loxP-spec-loxP mYPet-dnaE*	Gift of Joseph Loparo (Harvard Medical School); strain ET121	—
EST089	Halo-DnaE	PY79 *loxP-spec-loxP halo-dnaE*	Gift of Joseph Loparo (Harvard Medical School); strain ET138	—
EST101	*E. coli* prototrophic wild-type strain	MG1655	—	—
EST111	ΔPol Y1	PY79 *yqjH::loxP-spec-loxP*	EST111	(8)

EST181 (BWX5235)	Halo-DnaE^{AMGLA}	PY79 *loxP-spec-loxP halo-dnaE*^{AMGLA} *loxP-kan-loxP*	Transformation: *dnaE*^{AMGLA} *loxP-kan-loxP* → EST089	This study
EST183 (BWX5237)	Halo-DnaE^{QLSLF}	PY79 *loxP-spec-loxP halo-dnaE*^{QLSLF} *loxP-kan-loxP*	Transformation: *dnaE*^{QLSLF} *loxP-kan-loxP* → EST089	This study
EST185 (BWX5240)	Halo-DnaE^{AMGLA} DnaX-mYPet	PY79 *loxP-spec-loxP halo-dnaE*^{AMGLA} *loxP-kan-loxP dnaX-mYpet cat Ω pWX340a*	Transformation: EST181 → EST053	This study
EST187 (BWX5242)	Halo-DnaE^{QLSLF} DnaX-mYPet	PY79 *loxP-spec-loxP halo-dnaE*^{QLSLF} *loxP-kan-loxP dnaX-mYpet cat Ω pWX340a*	Transformation: EST183 → EST053	This study
EST203	Halo-DnaE DnaX-mYPet	PY79 *loxP-spec-loxP halo-dnaE dnaX-mYpet cat Ω pWX340a*	Transformation: EST089 → EST053	This study
EST205	*E. coli* protein expression strain	BL21(DE3)	—	—
EST281	DnaX-mYPet (ectopic)	PY79 *yvbJ::dnaX-mYpet cat*	Transformation: *yvbJ:dnaX-mYPet cat* → EST003	This study
EST283 (BWX5908)	PolC^{QMGLF}	PY79 *polC*^{QMGLF} *loxP-spec-loxP*	Transformation: *polC*^{QMGLF} *loxP-spec-loxP* → EST003	This study
EST285 (BWX5934)	PolC^{QMGLF}-mYPet	PY79 *polC*^{QMGLF}*-mYPet cat*	Transformation: *polC*^{QMGLF}*-mYPet cat* → EST003	This study
EST287	PolC-Halo DnaX-mYPet (ectopic)	PY79 *polC-halo loxP-spec-loxP yvbJ::dnaX-mYpet cat*	Transformation: EST083 → EST281	This study

Table S4. MALDI mass spectrometry characterization of CBM peptides. FITC = fluorescein-5-thiocarbamoyl, FAM = fluorescein-5(6)-carbonyl, Ahx = 6-aminohexanoyl; -OH indicates a C-terminal carboxylic acid; -NH_2 indicates a C-terminal amide.

CBM	Peptide Name	Full Sequence	Expected *m/z* ($M+H^+$)	Observed *m/z* ($M+H^+$)
QLSLPL	NSC1,1	FITC-Ahx-PAQLSLPLYL-NH_2	1615.8	1615.9
QADMA	NSC2,1	FITC-Ahx-IGQADMAGV-NH_2	1362.5	1362.1
QLSLF (PolC context)	PolC	FAM-Ahx-DQNQLSLF-OH	1435.6	1435.9
QMGLF	AC10B	FAM-Ahx-DDQMGLFLDE-NH_2	1652.6	1653.0
AMGLA	AC11B	FAM-Ahx-DDAMGLALDE-NH_2	1519.6	1520.1
QLSLF (DnaE context)	AC12B	FAM-Ahx-DDQLSLFLDE-NH_2	1664.7	1665.0

Table S5. Imaging dataset size.

Dataset/Condition	Figure(s)	Number of Days	Number of Replicates	Number of Cells	Number of Tracks or Foci
$DnaE^{QMGLF(WT)}$ D^*	4C, S5A	8	8	2,391	21,192
$DnaE^{AMGLA}$ D^*	4C	6	6	2,076	16,808
$DnaE^{QLSLF}$ D^*	4C	6	6	1,849	11,226
$PolC^{QLSLF(WT)}$ D^*	4C	3	4	959	14,522
$DnaE^{QMGLF(WT)}$-DnaX $g(r)$	4D	8	8	2,391	21,753
$PolC^{QLSLF(WT)}$-DnaX $g(r)$	4D	3	4	959	14,987
$DnaE^{AMGLA}$-DnaX $g(r)$	4D	6	6	2,076	17,843
$DnaE^{QLSLF}$-DnaX $g(r)$	4D	6	6	1,849	11,909
$PolC^{QLSLF(WT)}$-mYPet cellular localization	5C	3	3	482	823
$PolC^{QMGLF}$-mYPet cellular localization	5C	3	3	671	1,194
False positive spots D^*	S5B	3	3	724	84
False positive spots localization	S5B	3	3	724	93
DnaX cellular localization<br>Endogenous fusion	S6A	3	3	724	1,175
DnaX cellular localization<br>Ectopic fusion	S6B	2	2	711	991
DnaX cellular localization<br>Ectopic fusion + $PolC^{QMGL(WT)}$-Halo	S6C	3	4	959	1,357
DnaX cellular localization<br>Endogenous fusion + Halo-$DnaE^{QMGLF(WT)}$	S6D	8	8	2,391	3,813
DnaX cellular localization<br>Endogenous fusion + Halo-$DnaE^{AMGLA}$	S6E	6	6	2,076	3,539
DnaX cellular localization<br>Endogenous fusion + Halo-$DnaE^{QLSLF}$	S6F	6	6	1,849	2,281
$DnaE^{QMGLF(WT)}$ jump lengths	S7	8	8	2,391	88,030
$DnaE^{AMGLA}$ jump lengths	S7	6	6	2,076	75,237
$DnaE^{QLSLF}$ jump lengths	S7	6	6	1,849	53,824
$PolC^{QLSLF(WT)}$ jump lengths	S7	3	4	959	61,859
Halo-$DnaE^{QMGLF(WT)}$ cellular localization	S8A	8	8	2,391	23,138
Halo-$DnaE^{QLSLF}$ cellular localization	S8B	6	6	1,849	12,474
Halo-$DnaE^{AMGLA}$ cellular localization	S8C	6	6	2,076	18,472
$PolC^{QLSLF(WT)}$-Halo cellular localization	S8D	3	4	959	15,971

Table S6. Doubling times (mean ± standard deviation) for WT and PolC and DnaE CBM mutant strains for growth shaking at 225 rpm at 37 °C in minimal $S7_{50}$-sorbitol media and rich LB Lennox media. (Note: * indicates statistically significant difference at the $p < 0.05$ level relative to WT for same growth condition.)

**Strain / Media**	**WT**	**DnaE-AMGLA**	**DnaE-QLSLF**	**PolC-QMGLF**
$S7_{50}$-Sorbitol (min)	60 ± 1	58 ± 1	59.1 ± 0.6	58 ± 2
LB Lennox (min)	31 ± 1	28 ± 2 *	30 ± 1	41 ± 3 *

Table S7. Marker frequency analysis (MFA) of average *ori:ter* ratio (mean ± standard deviation) for WT and PolC and DnaE CBM mutant strains for growth shaking at 225 rpm at 37 °C in minimal $S7_{50}$-sorbitol media and rich LB Lennox media.

**Strain / Media**	**WT**	***dnaE*** ^{AMGLA}	***dnaE*** ^{QLSLF}	***polC*** ^{QMGLF}	***ssb D35***
$S7_{50}$-Sorbitol	1.65 ± 0.07	1.75 ± 0.07	1.80 ± 0.00	1.75 ± 0.07	2.20 ± 0.00
LB Lennox	3.60 ± 0.00	3.70 ± 0.00	3.75 ± 0.07	6.1 ± 0.7	5.7 ± 0.1

Table S8. Mutagenesis for different strains in untreated cells or cells treated with 40 J/m^2 UV light measured by the rate of rifampicin resistance (Rif^R) (mean ± standard deviation). (Note: * indicates statistically significant difference at the $p < 0.05$ level relative to WT for same treatment condition. All differences between untreated and treated for the same strain are statistically significant at the $p < 0.05$ level.)

**Strain**	**WT**		***dnaE*** ^{AMGLA}		***dnaE*** ^{QLSLF}	
**Condition**	**Untreated**	**Treated**	**Untreated**	**Treated**	**Untreated**	**Treated**
**Rif^R (per 10^8)**	0.7 ± 0.2	15 ± 5	0.9 ± 0.8	22 ± 10	1.1 ± 0.5	43 ± 5 *

Table S9. Doubling times (mean ± standard deviation) for WT and imaging strains for growth shaking at 225 rpm at 37 °C in minimal $S7_{50}$-sorbitol media. (Note: * indicates statistically significant difference at the $p < 0.05$ level relative to WT for same growth condition.)

**Strain**	**Doubling Time (min)**	**Strain**	**Doubling Time (min)**
WT	59 ± 2	$DnaE^{QMGLF(WT)}$-mYPet	101 ± 23 *
Halo-$DnaE^{QMGLF(WT)}$ DnaX-mYPet	58 ± 3	mYPet-$DnaE^{QMGLF(WT)}$	57 ± 4
$PolC^{QLSLF(WT)}$-Halo DnaX-mYPet	58 ± 4	$PolC^{QLSLF(WT)}$-mYPet	59 ± 2
		$PolC^{QMGLF}$-mYPet	61 ± 1

Table S10. Fold-change in number of colony-forming units (CFUs) per mL (mean ± standard deviation) in JFX_{554}-labeled vs. mock-labeled cultures after 1 h incubation. (JFX_{554} concentrations: 1 nM for Halo-DnaE, 500 pM for PolC-Halo).

**Strain**	**Fold-Change in CFUs/mL**	
	**Mock-Labeled**	**Labeled**
Halo-DnaE DnaX-mYPet	2.6 ± 0.5	2.7 ± 0.7
PolC-Halo DnaX-mYPet	2.1 ± 0.2	2.3 ± 0.5

Table S11. Cell length and width and number of DnaX or PolC foci per cell (mean ± standard error of the mean (S.E.M.)) (Note: ‡ indicates difference was not statistically significant at the $p < 0.05$ level. For DnaE and PolC HaloTag fusions, the comparison is made to the corresponding strain with the DnaX-mYPet fusion alone; for $PolC^{QMGLF}$-mYPet, the comparison is made to $PolC^{QLSLF(WT)}$. For DnaX-mYPet (ectopic), the comparison is made to DnaX-mYPet (endogenous).)

**Strain**	**Cell Length (μm)**	**Cell Width (μm)**	**Number of Foci per Cell**
DnaX-mYPet (endogenous)	3.09 ± 0.03	0.641 ± 0.003	1.62 ± 0.03
DnaX-mYPet (ectopic)	2.95 ± 0.03	0.589 ± 0.002	1.39 ± 0.03
Halo-$DnaE^{QMGLF(WT)}$ DnaX-mYPet	3.04 ± 0.02	0.665 ± 0.001	1.60 ± 0.02 ‡
Halo-$DnaE^{AMGLA}$ DnaX-mYPet	3.22 ± 0.02	0.686 ± 0.002	1.71 ± 0.02
Halo-$DnaE^{QLSLF}$ DnaX-mYPet	3.17 ± 0.02	0.650 ± 0.002	1.56 ± 0.02
$PolC^{QLSLF(WT)}$-Halo DnaX-mYPet	3.75 ± 0.03	0.636 ± 0.002	1.42 ± 0.03 ‡
$PolC^{QLSLF(WT)}$-mYPet	3.58 ± 0.04	0.641 ± 0.003	1.71 ± 0.04
$PolC^{QMGLF}$-mYPet	4.36 ± 0.04	0.611 ± 0.003	1.78 ± 0.04 ‡

Table S12. Halo-DnaE and PolC-Halo diffusion coefficient distribution fit parameters from MSD analysis (± uncertainties from 95% fit confidence intervals).

**Strain**	D_1 **(μm²/s)**	A_1	D_2 **(μm²/s)**	A_2	D_3 **(μm²/s)**	A_3
Halo-$DnaE^{QMGLF(WT)}$	0.084 ± 0.004	0.174 ± 0.011	0.306 ± 0.035	0.161 ± 0.020	1.0981 ± 0.040	0.666 ± 0.031
Halo-$DnaE^{AMGLA}$	0.082 ± 0.004	0.162 ± 0.011	0.335 ± 0.048	0.149 ± 0.025	1.125 ± 0.048	0.689 ± 0.036
Halo-$DnaE^{QLSLF}$	0.077 ± 0.005	0.205 ± 0.019	0.256 ± 0.040	0.157 ± 0.024	1.081 ± 0.055	0.637 ± 0.043
$PolC^{QLSLF(WT)}$-Halo	0.084 ± 0.004	0.186 ± 0.012	0.294 ± 0.034	0.190 ± 0.025	0.878 ± 0.036	0.624 ± 0.037

Table S13. Halo-DnaE and PolC-Halo diffusion coefficients and populations from Spot-On analysis.

**Strain**	D_{static} **(μm²/s)**	A_{static}	D_{free} **(μm²/s)**	A_{free}
Halo-DnaE$^{QMGLF(WT)}$	0.029	0.217	0.959	0.783
Halo-DnaE^{AMGLA}	0.025	0.198	0.982	0.802
Halo-DnaE^{QLSLF}	0.022	0.261	0.975	0.739
PolC$^{QLSLF(WT)}$-Halo	0.027	0.243	0.800	0.757

Table S14. Value of the mean radial distribution function $g(r)$ for DnaE-DnaX or PolC-DnaX colocalization at the second smallest value of r (the maximum of the $g(r)$ curve) and the standard error of the mean (S.E.M.) at that r value for the 100 calculated $g(r)$ curves.

**Strain**	$g(r)$ **± S.E.M.**
Halo-DnaE$^{QMGLF(WT)}$	1.272 ± 0.005
PolC$^{QLSLF(WT)}$-Halo	2.82 ± 0.02
Halo-DnaE^{AMGLA}	1.633 ± 0.007
Halo-DnaE^{QLSLF}	2.91 ± 0.02

Supplementary Methods

Overview of strain construction strategy:

Plasmids were constructed using standard molecular biology methods. Introduction of chromosomal genetic modifications by transformation of genomic DNA or double-stranded DNA (dsDNA) fragments assembled via Gibson assembly(9) was performed as described previously.(8) Tables S1 – S3 list all oligonucleotides, plasmids, and bacterial strains used in this study. Construction details for all new strains are summarized below.

Expression plasmids for *E. coli* and *B. subtilis* DnaN were cloned in the pET-28b vector. Constructs contained an N-terminal hexahistidine tag (His_6) and short flexible linkers (MGSSHHHHHHSSG) preceding the N-terminus of DnaN.

A weak-binding *polC* allele (*polC*^{QMGLF}) was generated by mutating the wild-type (WT) clamp-binding motif (CBM), QLSLF, to the *dnaE* CBM, QMGLF. Tight-binding and binding-deficient *dnaE* alleles were generated by mutation of the WT CBM, QMGLF, to the PolC CBM, QLSLF, and the double-alanine mutant, AMGLA, respectively (yielding the *dnaE*^{QLSLF} and *dnaE*^{AMGLA} alleles).

PolC and DnaE fusions to the self-labeling HaloTag(10) were constructed for single-molecule fluorescence microscopy. A C-terminal PolC-Halo fusion was built with an 11 amino acid linker (GSGQGSGPGSG) between the PolC C-terminus and the HaloTag N-terminus. Halo-DnaE constructs for microscopy were N-terminal fusions with an 8 amino acid linker (LEGSGQGP). Halo-DnaE fusions bearing the tight-binding and binding-deficient CBM mutations were also constructed. As a replisome marker, the clamp-loader complex subunit DnaX was fused at its C-terminus to the yellow fluorescent protein (YFP) variant mYPet(11) with an 8 amino acid linker (LEGSGQGP). Because the PolC-Halo fusion was synthetically lethal in combination with a DnaX-mYPet fusion at the endogenous *dnaX* locus, we created an ectopic DnaX-mYPet fusion introduced at the locus of the non-essential gene *yvbJ*; this gene plays no known role in replication. Replisomes in this merodiploid strain are expected to contain a mix of tagged and untagged DnaX molecules. Addition of the PolC-Halo fusion to this strain was tolerated, consistent with prior reports of strains bearing endogenous PolC fusions and ectopic DnaX fusions.(12, 13)

Although the PolC-Halo fusion was synthetically lethal with the *polC*^{QMGLF} weak-binding CBM mutation, a corresponding C-terminal mYPet fusion was viable. Therefore, PolC-mYPet and PolC^{QMGLF}-mYPet fusions were also constructed for microscopy. These fusions contained the 8 aa linker (LEGSGQGP).

Detailed plasmid construction information:

pEST007: pET-28b-His_6-Ec-DnaN. The pET-28b backbone was amplified from plasmid pEST005 using oligonucleotides oEST086 and oEST087. The *E. coli dnaN* gene was amplified

from strain EST101 using oligonucleotides oEST082 and oEST083. The two fragments were joined by Gibson assembly and transformed into strain EST001.

pEST009: pET-28b-His$_6$-Bs-DnaN. The pET-28b backbone was amplified from plasmid pEST005 using oligonucleotides oEST086 and oEST087. The *B. subtilis dnaN* gene was amplified from strain EST003 using oligonucleotides oEST084 and oEST085. The two fragments were joined by Gibson assembly and transformed into strain EST001.

Detailed strain construction information:

BWX2231: PolC WT loxP-spec-loxP. The C-terminal region of *polC* (including the stop codon) was amplified from strain EST003 using oligonucleotides oWX916 and oWX919, the *loxP-spec-loxP* cassette was amplified from plasmid pWX466 using universal primers oWX438 and oWX439, and the region downstream of the *polC* gene was amplified from strain EST003 using oligonucleotides oWX920 and oWX921. The three fragments were joined by Gibson assembly and transformed into strain EST003. This process results in the insertion of the *loxP-spec-loxP* cassette downstream of the WT *polC* gene.

EST007: SSB Δ35. The region containing the upstream of *ssb* and the ssb gene (without the final 35 amino acids) was amplified from strain EST003 using oligonucleotides oWX2235 and oWX2238, the *loxP-kan-loxP* cassette was amplified from plasmid pWX470 using universal primers oWX438 and oWX439, and the region downstream of the *ssb* gene was amplified from EST003 using oligonucleotides oWX2239 and oWX2240. The three fragments were joined by Gibson assembly and transformed into strain EST003. This process results in the deletion of 35 amino acids from the C-terminus of SSB, which is linked with the *loxP-kan-loxP* cassette.

EST009: DnaE loxPkan-loxP. The C-terminal region of *dnaE* (including the stop codon) was amplified from strain EST003 using oligonucleotides oWX900 and oWX903, the *loxP-kan-loxP* cassette was amplified from plasmid pWX470 using universal primers oWX438 and oWX439, and the region downstream of the *dnaE* gene was amplified from strain EST003 using oligonucleotides oWX904 and oWX905. The three fragments were joined by Gibson assembly and transformed into strain EST003. This process results in the insertion of the *loxP-kan-loxP* cassette downstream of the WT dnaE gene.

EST013: DnaE^{AMGLF}. The upstream and N-terminal part of *dnaE* was amplified from strain EST003 using oligonucleotides oWX900 and oWX2241, and the region containing the C-terminal part of *dnaE*, the *loxP-kan-loxP* cassette, and the downstream of *dnaE* was amplified from strain EST009 using oligonucleotides oWX905 and oWX2242. The two fragments were joined by Gibson assembly and transformed into strain EST003. This process results in the introduction of the *dnaE*^{AMGLF} CBM mutation.

EST043: DnaE^{AMGLA}. The upstream and N-terminal part of *dnaE* was amplified from strain EST013 using oligonucleotides oWX900 and oWX2354, and the region containing the C-terminal part of *dnaE*, the *loxP-kan-loxP* cassette, and the downstream of *dnaE* was amplified from strain EST013 using oWX905 and oWX2355. The two fragments were joined by Gibson assembly and transformed into strain EST003. This process results in the introduction of the *dnaE*^{AMGLA} CBM mutation.

EST047: DnaE^{QLSLF}. The upstream and N-terminal part of *dnaE* was amplified from strain EST003 using oligonucleotides oWX900 and oWX2249, and the region containing the C-terminal part of *dnaE*, the *loxP-kan-loxP* cassette, and the downstream of *dnaE* was amplified from strain EST009 using oligonucleotides oWX905 and oWX2357. The two fragments were joined by Gibson assembly and transformed into strain EST003. This process results in the introduction of the *dnaE*^{QLSLF} CBM mutation.

EST053: DnaX-mYPet. Plasmid pWX340 was transformed into strain EST003 and integrated into the chromosome by single crossover.(4) This process results in the introduction of the DnaX-mYPet fusion.

EST057: PolC-mYPet. The C-terminal domain of *polC* (without the stop codon) was amplified from strain EST003 using oligonucleotides oWX225 and oWX323, and the downstream region of *polC* was amplified from strain EST003 using oligonucleotides oWX324 and oWX228. These two PCR products were used as primers to amplify the *mYpet cat* allele from plasmid pWX318 according to the long-flanking-homology (LFH) method.(14) The final PCR product was transformed into strain EST003. This process results in the introduction of the PolC-mYPet fusion.

EST181: Halo-DnaE^{AMGLA}. The DnaE^{AMGLA} construct, the *loxP-kan-loxP* cassette, and the downstream region of *dnaE* were amplified from strain EST043 using oligonucleotides oWX900 and oWX905. The PCR product was transformed into strain ET089. The process creates the Halo-DnaE^{AMGLA} fusion.

EST183: Halo-DnaE^{QLSLF}. The DnaE^{QLSLF} construct, the *loxP-kan-loxP* cassette, and the downstream region of *dnaE* were amplified from strain EST047 using oligonucleotides oWX900 and oWX905. The PCR product was transformed into strain ET089. The process creates the Halo-DnaE^{QLSLF} fusion.

EST185: Halo-DnaE^{AMGLA} DnaX-mYPet. The *loxP-spec-loxP halo-dnaE*^{AMGLA} allele was transferred from strain EST181 to strain EST053 by transformation with genomic DNA. This process creates a strain bearing both the Halo-DnaE^{AMGLA} and DnaX-mYPet fusions.

EST187: Halo-DnaE^{QLSLF} DnaX-mYPet. The *loxP-spec-loxP halo-dnaE^{QLSLF}* allele was transferred from strain EST183 to strain EST053 by transformation with genomic DNA. This process creates a strain bearing both the Halo-DnaE^{QLSLF} and DnaX-mYPet fusions.

EST203: Halo-DnaE DnaX-mYPet. The *loxP-spec-loxP halo-dnaE* allele was transferred from strain EST089 to strain EST053 by transformation with genomic DNA. This process creates a strain bearing both the Halo-DnaE and DnaX-mYPet fusions.

EST281: DnaX-mYPet (ectopic). The N-terminal 500 bp of *yvbJ* and the upstream region were amplified from strain EST003 using oligonucleotides oEST118 and oEST119. The *dnaX-mYPet* fusion and the chloramphenicol cassette, including 150 bp of the *dnaX* upstream, were amplified from strain EST053 using oligonucleotides oEST120 and oEST121. The C-terminal 500 bp of *yvbJ* and the downstream were amplified from strain EST003 using oligonucleotides oEST122 and oEST123. The three fragments were joined by Gibson assembly and transformed into strain EST003. This process results in the introduction of the DnaX-mYPet fusion at the ectopic *yvbJ* locus.

EST283: PolC^{QMGLF}. The C-terminal region of *polC* (excluding the final 5 amino acids) was amplified from strain EST003 using oligonucleotides oWX916 and oWX3633, and the linker, *loxP-spec-loxP* cassette, and the region downstream of *polC* were amplified from strain BWX2231 using oligonucleotides oWX921 and oWX3634. The two fragments were joined by Gibson assembly and transformed into strain EST003. This process results in the introduction of the *polC^{QMGLF}* CBM mutation.

EST285: PolC^{QMGLF}-mYPet. The region upstream of the *polC* gene was amplified from strain EST003 using oligonucleotides oWX916 and oWX3633, and the linker, mYPet, cat cassette, and region downstream of *polC* were amplified from strain EST057 using oligonucleotides oWX921 and oWX3692. The two fragments were joined by Gibson assembly and transformed into strain EST003. This process creates a strain bearing the PolC^{QMGLF}-mYPet fusion.

EST287: PolC-Halo DnaX-mYPet (ectopic). The *polC-halo loxP-spec-loxP* allele was transferred from strain EST083 to strain EST281 by transformation with genomic DNA. This process creates a strain bearing both the PolC-Halo and ectopic DnaX-mYPet fusions.

Supplementary References

1. Wing,R.A., Bailey,S. and Steitz,T.A. (2008) Insights into the Replisome from the Structure of a Ternary Complex of the DNA Polymerase III α-Subunit. *Journal of Molecular Biology*, **382**, 859–869.

2. Evans,R.J., Davies,D.R., Bullard,J.M., Christensen,J., Green,L.S., Guiles,J.W., Pata,J.D., Ribble,W.K., Janjic,N. and Jarvis,T.C. (2008) Structure of PolC reveals unique DNA binding and fidelity determinants. *Proc. Natl. Acad. Sci. U.S.A.*, **105**, 20695–20700.

3. Timinskas,K., Balvočiūtė,M., Timinskas,A. and Venclovas,Č. (2014) Comprehensive analysis of DNA polymerase III α subunits and their homologs in bacterial genomes. *Nucleic Acids Research*, **42**, 1393–1413.

4. Wang,X., Montero Llopis,P. and Rudner,D.Z. (2014) *Bacillus subtilis* chromosome organization oscillates between two distinct patterns. *Proc Natl Acad Sci U S A*, **111**, 12877–12882.

5. Wang,X., Le,T.B.K., Lajoie,B.R., Dekker,J., Laub,M.T. and Rudner,D.Z. (2015) Condensin promotes the juxtaposition of DNA flanking its loading site in *Bacillus subtilis*. *Genes Dev.*, **29**, 1661–1675.

6. Zeigler,D.R., Prágai,Z., Rodriguez,S., Chevreux,B., Muffler,A., Albert,T., Bai,R., Wyss,M. and Perkins,J.B. (2008) The Origins of 168, W23, and Other *Bacillus subtilis* Legacy Strains. *J Bacteriol*, **190**, 6983–6995.

7. Schroeder,J.W. and Simmons,L.A. (2013) Complete Genome Sequence of *Bacillus subtilis* Strain PY79. *Genome Announc*, **1**, e01085-13.

8. Marrin,M.E., Foster,M.R., Santana,C.M., Choi,Y., Jassal,A.S., Rancic,S.J., Greenwald,C.R., Drucker,M.N., Feldman,D.T. and Thrall,E.S. (2024) The translesion polymerase Pol Y1 is a constitutive component of the *B. subtilis* replication machinery. *Nucleic Acids Research*, **52**, 9613–9629.

9. Gibson,D.G., Young,L., Chuang,R.-Y., Venter,J.C., Hutchison,C.A. and Smith,H.O. (2009) Enzymatic assembly of DNA molecules up to several hundred kilobases. *Nat Methods*, **6**, 343–345.

10. Los,G.V., Encell,L.P., McDougall,M.G., Hartzell,D.D., Karassina,N., Zimprich,C., Wood,M.G., Learish,R., Ohana,R.F., Urh,M., *et al.* (2008) HaloTag: A Novel Protein Labeling Technology for Cell Imaging and Protein Analysis. *ACS Chem. Biol.*, **3**, 373–382.

11. Nguyen,A.W. and Daugherty,P.S. (2005) Evolutionary optimization of fluorescent proteins for intracellular FRET. *Nat Biotechnol*, **23**, 355–360.

12. Li,Y., Chen,Z., Matthews,L.A., Simmons,L.A. and Biteen,J.S. (2019) Dynamic Exchange of Two Essential DNA Polymerases during Replication and after Fork Arrest. *Biophysical Journal*, **116**, 684–693.

13. Hernández-Tamayo,R., Oviedo-Bocanegra,L.M., Fritz,G. and Graumann,P.L. (2019) Symmetric activity of DNA polymerases at and recruitment of exonuclease ExoR and of PolA to the *Bacillus subtilis* replication forks. *Nucleic Acids Research*, **47**, 8521–8536.

14. Wach,A. (1996) PCR-synthesis of marker cassettes with long flanking homology regions for gene disruptions in *S. cerevisiae*. *Yeast*, **12**, 259–265.
